# Complete Genome Sequence of Raoultella ornithinolytica Strain S12, a Lignin-Degrading Bacterium Isolated from Forest Soil

**DOI:** 10.1128/genomeA.00104-15

**Published:** 2015-03-19

**Authors:** Wenying Bao, Yun Zhou, Jingwei Jiang, Zhihui Xu, Liyuan Hou, Frederick Chi-Ching Leung

**Affiliations:** a Bioinformatics Centre, Nanjing Agricultural University, Nanjing, China; b School of Biological Sciences, University of Hong Kong, Hong Kong SAR, China

## Abstract

We report the complete genome sequence of Raoultella ornithinolytica strain S12, isolated from a soil sample collected from areas bordering rotten wood and wet soil on Mt. Zijin, Nanjing. The complete genome of this bacterium may contribute toward the discovery of efficient lignin-degrading pathways.

## GENOME ANNOUNCEMENT

Strain S12 was originally determined by the appearance of a decolorizing ring in the identification medium with Azure-B (Genview) (1) and then screened based on its ability to grow on minimal media with alkali lignin (Sigma) supplied as the sole carbon source under aerobic conditions. It was identiﬁed as Raoultella ornithinolytica by comparing its 16S rRNA gene sequence to the nucleotide database in NCBI using BLASTn, and the BLAST result showed the highest identity, 99%, to strain S1776 (NCBI accession number KJ599629) (2). The genus Raoultella (formerly Klebsiella) is facultatively anaerobic, having both a respiratory and a fermentative type of metabolism. Most strains produce acid, gas, or 2,3-butanediol as a major end product of glucose fermentation (3).

The genomic DNA of strain S12 was extracted using the Bacterial DNA kit (Omega) from a pure, aerobic culture in Luria broth. Its quality and quantity were, respectively, examined and measured using a NanoDrop2000 spectrophotometer (Thermo Scientiﬁc) and the Quant-iT PicoGreen dsDNA kit (Invitrogen). A 2 × 300 MiSeq library and a 454 8-kb paired-end library were constructed and sequenced with the Illumina MiSeq (Illumina) and 454 GS Junior (454 Life Sciences) platforms, respectively, at the Bioinformatics Centre, Nanjing Agricultural University. The Illumina MiSeq platform achieved 1,337,594 reads that were longer than 250 bp with a mean quality score above 30. The 454 GS Junior platform achieved 62,742 reads. All these reads were assembled, using the Newbler version 2.7 assembly software program (Roche), into 77 large contigs with a 130-fold coverage of 5,494,953 bases. Gaps between contigs were closed by long-range PCR, the products of which were sequenced by Sanger sequencing and subsequently assembled using SeqMan software (DNASTAR).

The complete genome of strain S12 was submitted to the NCBI Prokaryotic Genome Automatic Annotation Pipeline (PGAAP) for annotation. All predicted genes were translated into amino acid sequences by in-house Perl scripts. Their functional classifications were performed by aligning these sequences to the Clusters of Orthologous Groups (COGs) database, using BLASTp with *E* values of 1 × 10^−5^ and filtering at 20% match identity and 90% alignment length (4, 5). Metabolic pathways were analyzed by using the bidirectional best-hit (BBH) method on the KEGG Automatic Annotation Server (KAAS) (6).

Raoultella ornithinolytica strain S12 has one chromosome, which is 5,522,044 bp in size with a G+C content of 57.47%. The genome has a total of 5,075 genes, 4,875 predicted coding sequences (CDS), 86 pseudogenes, and 18 frameshifted genes. Some other features were identiﬁed, including 85 tRNAs, 25 rRNAs, 4 ncRNAs, and 1 CRISPR array. According to the BLASTp result, approximately 78.4% of the genes were assigned to speciﬁc COGs, and 52.6% were involved in 196 predicted metabolic pathways. It was found that several genes are related to metabolic pathways, such as the degradation of aromatic compounds, starch and sucrose metabolism, and methane metabolism, providing the basis for the lignin-degrading phenomenon.

### Nucleotide sequence accession number.

The complete genome sequence of Raoultella ornithinolytica strain S12 has been deposited in GenBank under the accession number CP010557 for the chromosome. The version described in this study is the first version.
